# Precision Agriculture for Crop and Livestock Farming—Brief Review

**DOI:** 10.3390/ani11082345

**Published:** 2021-08-09

**Authors:** António Monteiro, Sérgio Santos, Pedro Gonçalves

**Affiliations:** 1 Agrarian Superior School, Polytechnic Institute of Viseu, Quinta da Alagoa, 3500-606 Viseu, Portugal; sergioalvesstos@gmail.com; 2 CERNAS, Research Centre for Natural Resources, Environment and Society, Polytechnic Institute of Viseu, Polytechnic Campus, 3500-606 Viseu, Portugal; 3 Escola Superior de Tecnologia e Gestão de Águeda and Instituto de Telecomunicações, Campus Universitário de Santiago, Universidade de Aveiro, 3810-193 Aveiro, Portugal; pasg@ua.pt

**Keywords:** crop and animal production, smart farming technologies, precision agriculture, precision livestock farming, trends

## Abstract

**Simple Summary:**

Precision agriculture has the potential to contribute to the broader objective of meeting the growing demand for food, ensuring the sustainability of primary production, based on a more accurate and resource-efficient approach to crop and livestock management. The aim of this paper consists of a brief review of the recent scientific and technological tools and sensors in precision agriculture and their application in crop and livestock farming. This literature review allowed us to realize that precision agriculture has been proven to be a highly researched and constantly evolving area due to the needs of farmers to use resources in a more optimized way.

**Abstract:**

In the last few decades, agriculture has played an important role in the worldwide economy. The need to produce more food for a rapidly growing population is creating pressure on crop and animal production and a negative impact to the environment. On the other hand, smart farming technologies are becoming increasingly common in modern agriculture to assist in optimizing agricultural and livestock production and minimizing the wastes and costs. Precision agriculture (PA) is a technology-enabled, data-driven approach to farming management that observes, measures, and analyzes the needs of individual fields and crops. Precision livestock farming (PLF), relying on the automatic monitoring of individual animals, is used for animal growth, milk production, and the detection of diseases as well as to monitor animal behavior and their physical environment, among others. This study aims to briefly review recent scientific and technological trends in PA and their application in crop and livestock farming, serving as a simple research guide for the researcher and farmer in the application of technology to agriculture. The development and operation of PA applications involve several steps and techniques that need to be investigated further to make the developed systems accurate and implementable in commercial environments.

## 1. Introduction

Agriculture has played a key role in the global economy in recent years [1]. Estimates show that current agricultural production must increase 60–100 percent with everything else unchanged to meet the nutritional needs of a future human population of 9–10 billion. In addition, agricultural intensification over the last few decades has had negative environmental impacts [2]. As a result, the pressure on the agricultural system is greater than ever before [1]. In order to minimize these issues, traditional agricultural management methods have been complemented by new sensing and driving technologies and improved information and communication technologies (ICT) [3]. Based on the concept of “produce more with less” [4], precision agriculture, also known as precision farming or smart farming, has the potential to contribute to the wider goal of meeting the increasing demand for food whilst ensuring the sustainability of primary production, based on a more precise and resource-efficient approach to production management [5].

PA technologies are used in the important stages of the crop growth cycle (soil preparation, seeding, crop management, and harvesting). However, it is not just crop and fruit farming that has benefited from precision farming technologies—farmers engaged in livestock rearing are also experiencing the positive benefits derived from precision farming technologies [5]. PA could be divided into two categories: precision crop farming, which consists of the application of precision farming technologies to manage spatial and temporal variability for improving crop performance and environmental quality, and PLF, which is based on the use of advanced technologies to optimize the contribution of each animal. Through this “per animal” approach, the farmer aims to achieve better results in livestock farming [4]. Precision crop farming and PLF are currently being shaped by two major technological trends: big-data and advanced-analytics capabilities on the one hand, and aerial imagery, feeding and milking robots, and intelligent sensors, on the other [6].

The current paper aims to briefly review the recent scientific and technological trends of precision farming and its application in crop and livestock farming. This study can serve as a research guide for both the researcher and the farmer in applying technology to agriculture. The remainder of this paper is organized as follows. Section 2 presents precision crop farming including different farming activities such as soil monitoring, precision seeding, smart irrigation and fertilization, and grass yield monitoring. It is also an approach to farm machinery and its important role in precision farming. Section 3 presents the PLF and scientific and technological developments concerning animal behavior, welfare, feed and live weight measurement, and automatic milking systems. In Section 4, slight considerations are made based on the risks and concerns inherent to precision farming. Finally, in Section 4, we present the conclusions.

## 2. Precision Crop Farming

Precision crop farming or site-specific crop management is a concept based on sensing or observing and responding with management actions to spatial and temporal variability in crops. The “sensing” component of the concept is a fundamental element of precision crop farming [7]. Sensors in fields and crops are starting to provide granular data points on soil conditions as well as detailed information on climate, fertilizer requirements, water availability, and pest infestations. In addition, aerial images captured by non-aircraft vehicles, crews, or drones can patrol fields, alert farmers to crop maturation or potential problems, and provide early warning of deviations from expected growth rates or quality. Satellites can also be at the service of precision crop farming, facilitating the detection of relevant changes in the field by using satellite imagery [8]. In this section, we introduce some of the scientific research and technological developments involved with smart crop farming (Table 1) as well as the important role of machinery in precision farming.

### 2.1. Evaluation of Soil Properties by Sensor Measurements

There are a range of sensors available used to measure and calculate the parameters of an agricultural field, namely the soil sensors [9], which can save labor and be a useful management tool, often providing more timely results, if they are accurate and the data are correctly interpreted [10].

Soil electrical conductivity (ECa) sensors measure the soil solute concentration while assessing the soil salinity hazard [11]. Mobile measurements of ECa have become widely used to map soil variability. The greatest potential use of ECa scanning is in the survey of spatial soil variability and delineating potential site-specific management zones. This, in turn, would allow for better resource allocation and long-term management planning [10]. Soil water content sensors such as frequency domain reflectometry (FDR) or time domain reflectometry (TDR) sensors measure the amount of water (volume or mass) contained in a unit volume or mass of soil by using electrodes. It is expressed by the change in capacitance value, which depends on the dielectric constant of the soil. It can range from 0 (completely dry) to the value of the materials’ porosity at saturation [12]. The sensor must be calibrated for each location because the measurements depend on the type of soil [9]. Soil moisture content sensors (or “volumetric water content sensors”) such as tensiometers evaluate the soil water tension or suction, which is a denotation of the plant root system effort while extracting water from the soil. It can be used to estimate the amount of stored water in the soil or how much irrigation is required to reach a desired amount of water in the soil [13]. Soil moisture content can also be determined by Photodiode, an optical sensor that uses the light to measure soil properties, namely clay, organic matter, and moisture content of the soil [9]. Smolka et al. [14] recently presented a mobile sensor aimed for on-site analysis of soil sample extracts used to detect the primary plant nutrients in their available form, at a fraction of the time and cost associated with traditional laboratory soil analysis. The sensor was particularly appropriate for the analysis of NO_3_, NH_4_, K, and PO_4_ and followed on from previous studies exploring the potential for on the-go soil sampling for nitrate using electrochemical sensor platforms and ion-selective electrodes [10]. There are also other types of sensors such as ground penetrating radar (GPR) and gamma ray spectrometry (GRS), which can be used through the ground cover vegetation. GPR data were correlated with soil hydrology parameters, and GRS data were related to some soil nutrients and other soil texture characteristics. Sensors based on optical reflectance as well as multi-spectral and hyperspectral sensors also have good correlation with soil properties [15,16]. Additionally, researchers from the EU-funded MISTRALE project have designed a system that can measure soil moisture from a drone flying at low altitude using Global Navigation Satellite Systems (GNSS) reflectometry. The system, still a prototype, can produce high-resolution maps of soil moisture by harnessing signals from either the Galileo or GPS global satellite systems. This could help farmers make better decisions about when and where to irrigate, and to help water managers understand weather events such as flooding and water logging [5].

### 2.2. Precision Seeding

Precision seeding has become the main feature and developing direction of modern seeding technologies in recent years. Precision seeding can save seeds and effectively control the sowing depth, densities, or distance. According to statistics, the output of precision seeding increases by 10–30% compared with that of the conventional drill [17]. Recent research has been carried out in order to make the sowing process more precise and efficient.

One of the most important aspects to achieve the consistent emergence of the crop desired is the use of a uniform seeding depth. Consistent crop emergence influences final crop yields, as the seedling growth process affects the variability of crop biomass. In this sense, Nielsen et al. [18] developed and studied a proof-of-concept dynamic coulter depth control system for a low-cost seed drill in a field experiment. The performance of the active control system was evaluated based on coulter depth measurements, obtained by coulter position sensors combined with ultrasonic soil surface sensors. The system was found to be capable of maintaining a more accurate and stable coulter depth across the field [18].

In light of the problem that small seeds are not easy for mechanized seeding at present, Jin et al. [19] developed and tested an electric seeder for small-size vegetable seeds based on the power drive and optical fiber detection technology. The system can perform precision seeding and real-time monitoring of the quality of seeding, and can complete the functions of furrow, seeding, and repression at a time, improving the quality of field operation and the accuracy of monitoring the seeding effect.

Due to the particular characteristics of the wheat seed grain geometry, Haibo et al. [20] designed and developed a wheel mobile robot for wheat precision seeding. A kinematic model was built for the four-wheel drive robot, and some experiments were taken using this machine. It provided a reference for the design and product of the wheat precision seeding robot. The experiment results showed that the qualified rates of seeding exceeded 93% at different sowing speeds.

Jianbo et al. [17] designed a control system based on a single chip microcomputer that could make the seed-metering device maintain synchronization with the working speed of the seeder. The experimental results proved that the control system was reliable and also showed that uniformity could be maintained between the speed of the seed metering-device and the seeder concerning the amount of seeding.

A high-speed precision seeding device enables one to obtain better seeding quality. This system consists of a seed supply device (including a seed box and a wheel) and a venture tube. In this sense, Gao et al. [21] designed a new air-assisted high speed precision seed metering device that could solve the problems of short filling time during high-speed operation and reduce the accumulation of seeds in the venturi tube. The results proved that for better design of the quantitative seed feeding system, the proper nozzle convergence angle is 70°, and the feeding angle around 45°.

### 2.3. Smart Irrigation Systems

The efficient and effective management of water in irrigation is one of the main benefits that comes from precision agriculture technologies [4], being of critical importance for sustainable agricultural development, food security, and overall economic growth. This is particularly true considering climate changes and the competing demand for water from other economic sectors. Precision irrigation allows farmers to apply a precise amount of water to crops at precise times [5]. In fact, instead of traditional irrigation, increasing attention has been paid to knowledge-and technology-driven smart irrigation due to advantages such as automatic controllability and feasibility in optimizing crop yield and irrigation water use efficiency (IWUE) [22].

In recent years, the literature has provided several studies on the optimization of irrigation water management. Ortega et al. [23] evaluated the effects of different temperatures on greenhouse tomato growth using an automatic irrigation system to suggest an optimal irrigation strategy for improving the IWUE. Goap et al. [24] proposed an IoT based smart irrigation system along with a hybrid machine learning based approach to predict the soil moisture with very encouraging results. The proposed algorithm uses the sensors’ data of the recent past and the weather forecasted data for the prediction of soil moisture for upcoming days. Furthermore, the prediction approach is integrated into a cost-effective standalone system prototype as it is based on open-source technologies. The auto mode makes it a smart system and it can be further customized for application specific scenarios.

In the south of Italy, Corbari et al. [25] developed an operational irrigation water management system using satellite LANDSAT data and meteo-hydrological modeling, which is based on the coupling of remote sensing data, a hydrological model of distributed water-energy, and meteorological forecasts. As a result, the authors concluded that operative applications of parsimonious irrigation are feasible by integrating data from satellites for updating and parameterizing the state of the hydrological model and meteorological forecasts, thus improving the management of irrigation scheduling.

Krishnan et al. [26] proposed a smart irrigation system to help farmers water their agricultural fields using Global System for Mobile Communication (GSM). According to the authors, this system provides a long-term sustainable solution for automatic irrigation control. Based on the data received from the soil moisture sensor, temperature sensor, and rain sensor, water is supplied to the agricultural field, helping in the consumption of water. By using GSM technology, the full system is automated to drastically reduce manual work [26].

Al-Ali et al. [27] developed an IoT-based renewable solar energy system important for regions that face water scarcity and power shortages around the world. This system uses a single board system-on-a-chip controller, which has built-in WiFi connectivity, and connections to a solar cell to provide the required operating power. The controller reads the field’s soil moisture, humidity, and temperature sensors, and emits actuation command signals to operate irrigation pumps.

Benyezza et al. [28] designed an intelligent and low-cost irrigation system based on zoning to make water use and energy consumption more efficient. This system consists of the collection and transmission of data on the soil and environmental conditions to a base station (server) using a wireless sensor network based on radio frequency communication. Since these data were collected and stored on a server, the time needed for irrigation in each zone was calculated using a fuzzy logic controller (FLC). When combining FLC with a zoning strategy, the system showed better results in terms of minimizing water and energy consumption when compared to other methods.

Recently, Liao et al. [29] developed a smart irrigation system based on real-time soil moisture data to estimate the dynamic crop water uptake depth (WUD) using the spatiotemporal characteristics of soil moisture distributions. According to the authors, this study estimated the tomato WUD from the distribution characteristics of soil moisture in the profile, providing a real-time and effortless method to determine the dynamic designed irrigation depth for guiding irrigation events.

### 2.4. Smart Fertilization Systems

Precise fertilization techniques are the future of agriculture, in which nutrients are supplied in a controlled way with minimized losses to the environment, caused by over-fertilization and leaching of microelements to deeper soil layers, ground water, and surface water [30].

As an important part of precision agriculture, variable-rate technology (VRT) offers an effective way to protect the environment and increase economic benefit while farming with site-specific fertilizer inputs. Song et al. [31] developed a variable-rate fertilizer control system applied to a UAV-based granular fertilizer spreader for automatically adjusting the fertilizer application rate based on a prescription map. The optimized metering apparatus showed high reliability in adjusting the rotational speed of a fluted roller motor to control the application rate as the specific relationship between them. The control system showed good stability with small error and rapid response whenever in a fixed-rate pattern application, where the target discharge rate changed unregularly or in a variable-rate pattern where the prescription map was given [31].

To reduce the labor workload and costs, thus increasing the efficiency of the organic fertilizer mixing process, Ishak et al. [32] developed an improved organic fertilizer mixer based on the Internet of Things technology, which can monitor the status of fertilizer production remotely, thus providing updates and alerts to the farmers. According to the authors, the automated organic fertilizer prototype provides operational cost savings of over five times when compared to current automated systems.

From another aspect, robots are perfectly suited to be used in agricultural operations, particularly in the spraying of fertilizer, which can carry large storage reservoirs, be operated safely and autonomously, and be deployed at a fraction of the costs compared to the traditional methods. In this sense, Ghafar et al. [33] developed a low-cost agricultural robot to spray fertilizers and pesticides in agriculture fields as well as for general crop monitoring. The prototype system is a two-wheeled robot that consists of a mobile base, a spraying mechanism, a wireless controller to control the movement of the robot, and a camera for crop health and growth monitoring. According to the authors, tests conducted on the agricultural robot prototype showed that it could perform as required under real-world usage scenarios.

Additionally, fertigation technology is recognized as a new agricultural technology with high-efficiency water and fertilizer control. Lin et al. [34] developed a framework for the IoT-based fertigation system in which both long-term and short-term planning were considered. Based on the framework, an integer linear programming model was developed to allocate limited resources among multiple crops with the goal of maximizing the economic profits and environmental benefits. The results confirmed that the optimization model can promote sustainable irrigation and fertilization management in precision agriculture by offering more economic and environmental benefits than empirical models. In addition, Bai and Gao [35] proposed an optimization method for the maize nitrogen (N) fertilizer schedule by combining a decision support system for agrotechnology transfer (DSSAT) model with a genetic algorithm, after calibration and validation of the DSSAT model. According to the authors, GA and the DSSAT model can promote the optimized fertilizer schedule.

### 2.5. Grass Yield Monitoring

Monitoring and predicting above ground biomass yield are of key importance for crop management. According to Beukes et al. [36], a 15% increase in farm profitability could be achieved by carrying out regular herbage measurements [37].

Traditional methods of measuring grass yield include destructive grass sampling coupled with weighing systems on mowers, trailers, or balers [38]. Optical sensing techniques have recently evolved and, according to Schaefer and Lamb [39], it is now possible to estimate herbage biomass levels by combining a measure of pasture canopy height using light detection and ranging (LiDAR) and pasture canopy reflectance measurements using active optical sensors. Additionally, the prediction accuracy of herbage mass from ultrasonic height measurements was promising and could be improved further by using spectral reflectance signatures in combination with the ultrasonic sensor [10].

Current developments in unmanned aerial vehicle-based (UAV) sensing systems allow for the acquisition of image data at a ultrahigh spatial resolution for important stages of plant phenological growth [11,38]. Furthermore, new photogrammetric software products support the analysis of such image data to produce 3D point clouds and digital surface models (DSMs). The analysis of multi-temporal DSMs supports the monitoring of sward or crop height development in high spatial resolution [39]. A research by Lussem et al. [40] evaluated the potential of sward height metrics derived from low-cost UAV image data to predict forage yield. According to the authors, dry biomass yield can be predicted using sward height derived from multi-temporal DSMs derived from low-cost UAV-based imaging with consistent results over three years. The on-going miniaturization and cost efficiency of sensors and platforms as well as powerful algorithms and computer hardware can open new paths to sustainable grassland management [38]. In addition, research by Oliveira et al. [41] showed that technology drone-based spectral remote sensing and photogrammetry could be used to predict the yield with an adequate degree of accuracy and to study the spatial differences within a field and between different fields, thus indicating great potential for remote sensing and drones to support the management of silage production for animal feeding [41].

The capacity to yield-map crops is relevant and provides a decision support tool for improved crop management. An on-the-go pasture meter was developed in New Zealand to meet this need. The equipment uses optical sensors to determine sward height and needs to be calibrated for herbage density. It can be used as a stand-alone sward meter or with GPS for sward-yield mapping [42].

Finally, we should also refer to the grass measurement optimization tool (GMOT), which is designed to utilize basic pasture management and geo-spatial information to develop a spatially balanced and non-biased grass measurement protocol. Calibrations to predict herbage mass that were built into the GMOT could utilize herbage management information such as fertilization rates and the number of previously performed paddock grazing or cutting events to increase herbage mass prediction precision [37].

**Table 1:** Overview of precision crop farming technology and applications.

Reference	Application	Involved Technologies	Main Objective/Function
[10]	Soil management	Soil electrical conductivity sensor	Measures the soil solute concentration while assessing the soil salinity hazard
[12]	Soil management	Electrodes for frequency domain (FDR) or time domain reflectometry (TDR)	Measures soil water content
[13]	Soil management	Tensiometer	Detects the force used by the roots in water absorption
[9]	Soil management	Photodiode	Determines clay, organic matter andmoisture content of the soil
[14]	Soil management	Ion-selective electrodes (ISE) and ion-selective field effect transistor sensors (ISFET)	Used to detect the primary plant nutrients (NO3, NH4, K and PO4) in soils
[15]	Soil management	Ground penetrating radar (GPR) and gamma ray spectrometry (GRS)	GPR is related to soil hydrology parameters, and GRS data is related to some soil nutrients and other soil texture characteristics
[5]	Soil management	GNSS reflectometry	Produce high-resolution maps of soil moisture by the use of drone flying at low altitude
[18]	Seeding management	Seed drill depth control system	Maintaining of an adequate and uniform seeding depth
[19]	Seeding management	Electric seeder for small-size vegetable seeds base on power drive and optical fiber detection technology	Perform precision seeding; real-time monitoring the quality of seeding; furrow, seeding and repression at a time
[20]	Seeding management	Wheel mobile robot for the wheat precision seeding	Wheat precision seeding
[17]	Seeding management	Control system for seed-metering device using a single chip microcomputer	Make the seed-metering device keep synchronization with the working speed of the seeder
[21]	Seeding management	Air-assisted high speed precision seed metering device	Solve short filling time issues during high-speed operation; reduce the accumulation of seeds in the venturi tube
[23]	Water management	Automatic irrigation system	Optimal irrigation strategy for improving the irrigation water use efficiency
[24]	Water management	IoT based smart irrigation system along with a hybrid machine learning based approach	Predict the soil moisture
[25]	Water management	Water management system using satellite LANDSAT data and meteo-hydrological modeling	Development of an operational irrigation system for water management
[26]	Water management	Smart irrigation system using global system for mobile communication (GMS)	Help farmers water their agricultural fields
[27]	Water management	IoT-based renewable solar energy system	Appropriate actuation command signals to operate irrigation pumps
[28]	Water management	Low-cost irrigation system based on wireless sensor network using a radio frequency communication.	Make water use and energy consumption more efficient
[29]	Water management	Smart irrigation system based on real-time soil moisture data	Determine the dynamic designed irrigation depth for guiding irrigation events
[31]	Fertilizer management	Variable-rate fertilizer control system based on ZigBee technology	Automatically adjust the fertilizer application rate based on a prescription map
[32]	Fertilizer management	Improved organic fertilizer mixer based on the Internet of Things (IoT)	Monitoring the status of fertilizer production remotely providing updates and alerts to the farmers
[33]	Fertilizer management	Low-cost agricultural robot (prototype)	Spray fertilizers safely and autonomously; general crop monitoring
[34]	Fertilizer management	IoT-based fertigation system	Promote sustainable irrigation and fertilization management offering more economic and environmental benefits than empirical models
[35]	Fertilizer management	Model based on decision support system for agrotechnology transfer (DSSAT) and genetic algorithm	Used to optimize the nitrogen fertilizer schedule of maize under drip irrigation
[39]	Grass yield management	LiDAR plant height detecting sensor integrated with an active optical NDVI sensor	Estimate of green fraction of biomass in swards comprising both senescent and green material
[10]	Grass yield management	Spectral reflectance signatures in combination with the ultrasonic sensor	Prediction accuracy of herbage mass from ultrasonic height measurements
[38]	Grass yield management	Unmanned aerial vehicle-based (UAV)	Acquisition of image data in ultrahigh spatial resolution for important phenological growth stages
[40]	Grass yield management	Low-cost UAV-based imaging	Prediction of forage yield
[41]	Grass yield management	Drone-based imaging spectrometry andphotogrammetry	Managing and monitoring of quantity and quality of grass swards used for silage production
[42]	Grass yield management	On-the-go pasture meter using optical sensors and GPS	Used as a stand-alone sward meter or sward-yield mapping
[37]	Grass yield management	Grass measurement optimization tool (GMOT)	Development of a spatially balanced and non-biased grass measurement protocol using basic pasture management and geo-spatial information

### 2.6. Linking Technology to Farm Machinery

Mechanization is one of the main drivers of efficient farming systems, involving numerous operations in the crop’s production cycle and throughout the value chain [43]. Agricultural mechanization started with steam powered reapers and traction engines, and then advanced with the invention of mobile hydraulics and the electronic control systems that are currently used in modern machinery [44]. Indeed, mechanized farming has been adopting increased levels of automation and intelligence to improve management and increase productivity in field operations. Nowadays, farmers can use, for example, auto-steered agricultural vehicles for many different field operations including tilling, planting, chemical applications, and harvesting. Intelligent machinery for automated thinning or precise weeding in vegetable and other crops has recently been introduced to farmers [45].

Recently, the accomplishment of agricultural operations carried-out with tractors has been heading toward the integration of traditional mechanics with geolocation technologies to bring farming procedures closer to the logic of precision agriculture. The possibility of controlling the tractor steering by using GNSS is an important contribution to improving the efficiency of agricultural practices and allows savings of time, fuel, labor, and production factors contributing to the economic and environmental sustainability of the agricultural process [46]. The automatic steering system or autopilot system tractor combines digital analysis and the image processing method, cameras, and GPS. In general, the system is comprised of two major elements that are hardware (various sensor and actuator such as GPS, a steering angle sensor) and software (path planner, a navigation control and steering controller). With high accuracy in the automated steering system, the farmer is able to automatically run the tractor throughout the desired path without causing any damage to the vegetation while maintaining a wide range of measurement [47]. 

The latest developments in ICT and the prevailing lack of interoperability between agricultural tractors, implements, and on-board computers has led to the development of the ISO 11783 (ISOBUS) international standard for securing a more effective communication between these entities. Precision agriculture requires an increasing amount of information in order to be sufficiently managed and the abilities of the ISOBUS protocol is a significant step toward this goal as it will provide a wealth of automated data acquisition for improving the management of crop production [48].

In addition, mechatronic systems can also play an essential role in modern agricultural machinery, especially on intelligent and robotic vehicles [45]. The term “agricultural robot” or “agrobot” applies to autonomous machines that are capable of performing different repetitive agricultural tasks on the farm without direct human intervention [49]. Agricultural robots allow the farmer to reduce inputs—pesticides, herbicides, and fertilizers—with positive implications for the environment. Mechanical weed control is already a reality; other functions under development include the microapplication of inputs and early detection of pests, which will considerably decrease, even eliminate, the need for inputs. Agrobots are also lighter than tractors with implements or specific equipment for spraying or harvesting and can thus alleviate problems associated with soil compaction and are able to access fields not suitable for heavy machinery (e.g., vineyards on slopes or land affected by wet conditions) [43].

Mechatronics enables the practical implementation of variable rate application (VRA) equipment for precision agriculture. As VRA technology is progressing quickly, intelligent applicators are becoming available commercially. A smart system can automatically adjust the number of inputs dispersed in response to needs, even allowing for the simultaneous use of different types of treatments, resulting in new ways of managing agricultural production. Thus, an intelligent VRA seeder can, for instance, change the number of seeds laid out in the soil according to its potential, either provided by prescription maps or detected using on-board sensors. Control of the seeding rate is achieved by actuating the opening of the distributing device to allow the desired number of seeds to go through. In many cases, a feedback control system is required to achieve accurate control of the application rate. For example, in applying liquid chemicals, the application rate may be affected by changes in the moving speed of the vehicle as well as the environmental conditions. Some smart sprayers are programmed to accurately control the amount of liquid chemical by adjusting the nozzles in response to changes of sprayer forward speed. This is normally accomplished using electronically controlled nozzle valves that are commanded from the on-board processor [45].

Today’s commercial farmer, who has a full command of the existing farming skills and knowledge, will need to become a sort of information technology (IT) manager operating from an office or in front of a screen (computer, mobile phone, tablet etc.), rather than a machine operator working in the field, handling machine steering, and adjusting equipment manually. For livestock management, skilled operators will still be needed, but with new sets of skills related to ICT and automatization [43].

## 3. Precision Livestock Farming

As part of precision farming, managing livestock is one of the current challenges for agriculture [50]. The term ‘precision livestock farming’ (PLF) appeared in the early 21st century, with the first PLF conference held in 2003 [51] as an innovative production system approach [52], playing a key role in the fourth industrial revolution, also known as Industry 4.0 [53]. PLF is potentially one of the most powerful developments amongst a few interesting new and upcoming technologies that have the potential to revolutionize the livestock farming industries [54].

PLF uses a combination of tools and methods to measure different variables from each animal with high precision, supporting farmers to make decisions concerning the livestock production systems [55]. Decisions are often based on the acquisition, collection, and analysis of quantitative data obtained by continuous real-time from animals and the environment [56,57]. These tools include sensor technology cameras [58], microphones, wireless communication tools, Internet connections, and cloud storage [59], among others. However, the application of the existing tools for PLF can be challenging under extensive livestock management because this occurs on natural pastures that are large, heterogeneous, and highly dynamic environments [55]. Therefore, the main purpose of PLF is to enhance farm profitability, efficiency, and sustainability [58,59] by improving on-farm acquisition, management, and utilization of data management and the utilization of data, in order to enhance the nutritional and other management aspects from distinct species of animals [59]. PLF could also deliver additional food safety, traceability, welfare, and environmental benefits [52,54,59,60]. In addition, PLF aims the management of crop processes to create perfect synergy with livestock feeding [3]. If properly implemented, PLF could (a) promote product segmentation and better marketing of livestock products; (b) reduce illegal trading of livestock products; and (c) improve the economic stability of rural areas [54]. In this section, we present some of the technological advancements and scientific research involved with PLF (Table 2).

### 3.1. Animal Monitoring

Successful grazing and pasture management require an understanding of the adjustment mechanisms behind the grazing behavior [61] that enables adaptation to grazing conditions [62]. As well as facilitating the precise management of grazing, the monitoring of animal position, foraging, and other behaviors can bring considerable benefits for animal health and welfare by continuously monitoring each animal in the flock, any small deviation from ‘normal’ behavior (for that individual animal) can be quickly identified and flagged to the farmer [63].

The use of GNSS technology allows for the characterization of grazing behavior including grazing patterns, paths, and favored areas. Grazing activities can also be differentiated based on the speed of movements. The increased knowledge conveyed using GNSS receptors in grazing sheep can become a valuable tool to support the decisions that are essential to a more precise pasture management [62].

Tracking location on pasture, through the large dissemination of global positioning system (GPS) sensors have been successfully used to detect static or dynamic unitary behaviors differentiated through changes in path speeds: foraging or grazing, resting, and walking [64]. Likewise, the use of GPS “collars” for livestock has opened the possibility of recording detailed position data for extended periods of time, thus allowing for a more complete understanding of the habits and causes of the spatial distribution of ruminants. Current GPS technology can determine the position of individual animals with a precision of 10 m or better. The position information can be stored on small flash cards together with substantial amounts of behavior and physiological data and can be transmitted to a management center in real time or in periodical sessions [65].

Behavior of cattle can also be monitored by means of a collar characterized by a behavior-monitoring series of sensors. Through analysis of rumination rate and the feeding and resting behavior, estrus events can be detected, according to a study by [58]. Similar collars were used, showing that the highest accuracy was achieved with these instruments (>90%) whereas visual human observation was far less accurate [66].

Posture analysis was developed using accelerometers and based on the position of the head: up or down. This information, in combination with GPS-based data, allowed for discrimination between several kinds of feeding related behaviors for grazing animals with high accuracies (>90%). These accuracies were obtained with a brief time window of 5 to 10 s while the data acquisition from the GPS and the accelerometer ran between 4 Hz and 10 Hz [67].

Finally, we monitored cattle movements using accelerometers. Through diverse analysis methods, accelerometers recording data at 10 Hz could be used to classify behaviors using a basic statistical method to classify lame and non-lame cows, reaching an average accuracy of 91% [64]. Similarly, in [67], they were classified as multiple behaviors using a machine learning method with accuracies ranging from 29% to 86% with samples windowed for 10 s for all behavior classifications.

Among the solutions to detect the animal behavior and collect data with a reduced uncertainty [68], image and sound analyses are also promising. However, video recordings require a large amount of time to be analyzed and manually checked, involving potential mismatches in the interpretation of observers. According to Meen et al. [69], there is a correlation between sounds and behavior, as a significant difference emerged between the average maximum frequency of murmurings during the lying and ruminating phase and that of calls during the other phases [66].

In addition, a review carried out by Meunier et al. [70] described machine learning algorithms such as pig-face recognition. Recent advancements in facial recognition have been extended to identify and recognize the patterns of several animal behaviors. Different facial detection and recognition methods such as the VGG-face model, Fisherfaces, and convolutional neural networks can now discern individual animal faces in complex real-time scenarios, in the presence of some shape deformation and even in instances where there is insufficient data. This non-invasive imaging system recognizes the faces of individual pigs in a real farm setting with 96.7% accuracy [71].

### 3.2. Animal Health and Welfare

Animal health is of key importance in the livestock industry as it impairs production efficiency through growth retardation or even mortality, animal welfare through pain and discomfort, and it can even impair human health through the misuse of antibiotics or zoonosis [72]. In fact, the large density of animals living so close to humans in some countries can transfer a high number of zoonosis diseases to humans [73]. The monitoring of health problems in the early detection of clinical signs of diseases on the farm is one of the key issues from which PLF has arisen [66]. Most diseases are easily treated when detected in an early phase, although prevention is always the priority [72]. Modern technologies such as sensors, big data, artificial intelligence (AI), and machine learning (ML) algorithms enable farmers to react to diseases after they become evident, or pro-actively using vet services, and also provide an opportunity to constantly monitor key animal health parameters such as movement, air quality, or consumption of feed and water. By constantly collecting these data and using advanced technology to predict deviations or abnormalities, farmers can identify, predict, and prevent disease outbreaks. Therefore, this technology has a significant cost advantage over older detection methods [71].

Animals can be monitored by methods based on the sound, with the potential to be automated for large-scale farming [70]. A sound-based tool (Pig Cough Monitor™ (PCM), Soundtalks^®^, and Fancom B.V.) has been developed for automated pig cough detection that is based on a mathematical algorithm that processes all incoming sound and identifies the number of coughs automatically [69]. In addition, Van Hertem et al. [74] evaluated the effect of using a microphone and subsequent advanced methods for labelling in the early detection of cough in calves and highlighted how the adoption of an algorithm with >90% precision allowed reducing the emergence of bovine respiratory disease (BRD). In addition, distress can be vocalized by animals or shown though unusual activity. Vocalization could be measured via microphones, whereas activity could be observed and recorded using staff observations or surveillance cameras, with the interpretation of sounds and images to produce meaningful information [75].

Nowadays, automated sensors and algorithms can reliably predict and reduce the risk of mastitis in cows. Air sensors in the poultry industry can predict the onset of Coccidiosis by constantly monitoring the concentration of volatile organic compounds in the air increase, as the number of infected birds increases. Air sensors could detect this change much earlier than a farmer or a vet could [71]. In other cases, by carrying out image analysis and calculating model parameters from the image information, it was possible to develop an algorithm for automatic detection of lameness based on animal locomotion [71,76]. In the case of cattle health, a few common diseases can be identified using non-invasive, cheap sensor technologies. More complex sensor platforms exist, for instance, camera systems to detect back posture, and ingestible pills for heart rate determination [77]. Furthermore, the continuous feed and water registration in the farm makes it possible to assess the first freedom from hunger and thirst. Climate control sensors such as temperature sensors, relative humidity probes, and CO_2_ sensors will allow the automatic evaluation of thermal discomfort in the house [72].

### 3.3. Feed and Live Weight Measurement

Precision feeding is a key component of PLF [60]. Based on real-time feedback from sensors [78], precision livestock feeding aims to provide to individuals or a group of animals with the amount of nutrients that maximizes its utilization without loss of performance [60]. Therefore, accurately and automatically measuring the amount of feed used per day per animal or distinct group of animals is extremely important [59]. The use of precision livestock feeding can decrease protein intake by 25%, and nitrogen excretion into the environment by 40%, while increasing profitability by nearly 10% [60].

The implementation of automated feeding systems (AFS) can provide a cost-effective alternative to manual regimes. Feeding units have been developed for a variety of animal systems including cattle, sheep, and pigs. These systems can be advantageous by providing an interface that monitors time and date of feeding, the electronic identification of each animal, the weight of the feed consumed, and the duration of feeding [79].

Demmers et al. [80] used an automated feeding system to control the amount of feed delivered to pens and the ambient temperature to optimize growth and reduce ammonia emissions [60]. In Australia, a feed sensor was developed to measure and control the amount of feed delivered to individual feeders quite precisely [59]. In Canada, a next generation feeding system was recently developed that will provide the additional capability to provide feed with a variety of nutrient specifications to tailor both the amount and composition of the feed. In the next few years, we might be able to adjust the nutrient intake to match the requirements of individual animals in real-time, based on their state-specific needs, as estimated from the sensor data [77]. We must also emphasize a study by Evangelista et al. [81] that highlights the use of portable near infrared spectroscopy (NIRS) to evaluate the physio-chemical composition of total mixed ration (TMR) and manure in dairy farms. According to the authors, the use on barn NIRS, through appropriate calibrations, is a rapid and accurate analytical technique with high potential benefits.

RGB-D cameras can also help farmers to measure feed intake for individual cows [82]. In addition, several advanced algorithms can help farmers calibrate and optimize feed expenses according to their animals’ needs [72]. Finally, mathematical nutrition models can be useful components to correctly estimate the contribution of ruminants to greenhouse gas emissions (GHG) [77].

The measurement of average live weight gain (speed of growth) of a distinct group of animals is one of the most important measurements to be undertaken on livestock farm as the speed of growth will affect both the financial performance of the farming enterprise as well as the final body composition of the animals [59]. Recent systems have appeared on the market (such as the Osborne Weight-Watcher™). Weighing systems based on image analysis techniques have been designed to determine the weight of individual or groups of animals (specifically pigs) with acceptable precision by correlating dimensional measurements of the animals to weight. Recent studies carried out by Banhazi et al. [82] have demonstrated that those systems can reliably provide a performance record of successive batches of animals and in a timely manner.

### 3.4. Automatic Milking Systems

The milking robot is a classical PLF application [50]. The growing popularity of this technology is evident in its rapid rate of adoption. Automatic milking systems (AMS) have gained widespread acceptance, particularly in western Europe to reduce labor on dairy farms, increase production per cow, and improve the lifestyle of dairy farm families [83]. The first installations were typically associated with ‘indoor’ systems and nearby grazing fields. Nowadays, there is an increasing interest regarding the integration of robots into larger scale pasture-based dairy systems. The milking process can now be spread over a 24-hour period, allowing the animal to choose when to be milked. The AMS can be applied both to indoor and pasture-based feeding systems [84].

There are numerous offers for automatic milking systems (AMSs) in the market. Recent technological advances have included the integration of more modern sensors/vision systems, the addition of animal monitoring features, and the integration of robots in rotary milking parlors. Time of flight (ToF) depth sensing cameras have been used in many recent developments in AMSs. Acceptable accuracy (teat location detection within 5 mm) has been achieved when the search space is limited to a region of interest 150 mm wide by 80 mm deep [85]. Teat detection and tracking using algorithmic solutions from depth images and point-cloud data were also achieved according a study by Rodenburg et al. [86]. Other vision technologies have also been investigated using a Kinect structured light camera and a Haar Cascade classifier [87], or using a combination of thermal imaging and stereovision techniques [88]. The task of attaching the milking clusters to the cows’ teats is a challenging one as the shape of the udder is variable between cows and between distinct stages of lactation [85].

Cycle time of the milking operation must also be minimal so that both the cow and farmer can be more productive with their time. Therefore, an intelligent control of the robot is required using visual feedback to navigate the cluster onto the cow accurately, safely, and quickly. A system that can milk cows with unusually shaped udders or that do not take to a robot milker, which would otherwise have to be culled as is the case with some existing AMSs, would also be a considerable advantage [85].

**Table 2:** Overview of PLF technology and applications.

Reference	Application	Involved Methods/Technologies	Main Objective/Function
[89]	Animal behavior	GPS sensors	Tracking location
[64]	Animal behavior	A neck collar with series of sensors	Detection of estrus events through analysis of rumination rate, and the feeding and resting behavior
[67]	Animal behavior	Accelerometers in combination with GPS-based data	Discrimination between several kinds of feeding related behaviors for grazing animals Classification of multiple cattle behaviors
[90]	Animal behavior	A machine learning method	Pig cough detection-processing all incoming sounds and automatically identifying the number of coughs
[69]	Animal behavior	Cameras and microphones Sound tool based on an algorithm	Find a correlation between vocalization and behavior
[71]	Animal behavior	A non-invasive imaging system such as VGG-face model, Fisherfaces, and convolutional neural networks	Pig-face recognition
[74]	Animal health and welfare	Microphones for cough sounds	Detect bovine respiratory disease
[70]	Animal health and welfare	Air sensors	Prediction the onset of Coccidiosis by monitoring the concentration of volatile organic compounds in the air
[76]	Animal health and welfare	Algorithm developed through image analysis	Automatic detection of lameness in dairy cows individually
[80]	Feed management	An automated feeding system	Control the amount of feed provided, and the ambient temperature to optimize animal growth and reduce ammonia emission
[58]	Feed management	A feed sensor	Measure and control the amount of feed delivered to individual feeders
[78]	Feed management	A next-generation feeding system	Provide feed with a variety of nutrient specifications to tailor both the amount and composition of the feed
[79]	Feed management	A computer vision based system CNN models using a low-cost RGB-D camera	Measures cow individual feed intake
[81]	Feed management	NIRS technology	Evaluation of physio-chemical composition of TMR and manure in dairy farms
[82]	Weight management	Weighing system based on image analysis	Determine the weight of individual or group of animals (specifically pigs)
[85,86,87,88]	Automatic milking systems	Time of flight (ToF) depth sensing cameras Algorithmic solutions from depth images and point-cloud data Machine learning based vision for smart MAS Combination of thermal imaging and stereovision techniques	Teat Detection Teat detection and tracking Capability for faster and accurate teat detection Teat sensing

## 4. Risks and Concerns about Precision Farming

It is important to understand how farmers interpret the value of technology in the context of their farms. On one hand, farmers look at value to their farming business in the adoption of the usage of new technologies to solve future problems [73]. On the other hand, many producers perceive that adopting high productive management systems involves increased risk [54]. The perceived risks involve the risk of financial failure because of unforeseen environmental or market circumstances, damage to the farm infrastructure such as soils and pasture, compromises to animal health and welfare, and the risk of increased stress on them from managing an intensified system [54,56]. Another risk that precision farming shares with other technologies is the further consolidation of farms as far as wealthier participants in a sector can benefit the most from recent technologies [91]. There is also the concern about some instances where technology cannot be used effectively. In some cases, farmers are either reluctant or may not be able to use the latest technology on their farms. The selling of pre-mature technology to farmers by companies without sufficient trials or evidence could result in costly losses for the farmers, namely, when it comes to predicting epidemic diseases in large scale animal farms. Furthermore, use of the data is itself a problem. Vast amounts of data from the technology products and services get stored in remote cloud servers. This is often monetized for commercial benefits. Big corporations can now collect, use, and even sell data from farmers. The rising tension between corporations and farmers over data misuse is a considerable threat [71].

## 5. Conclusions

With increasing population pressure around the world and the need to increase agricultural production, there is a concern for improved management of the world’s agricultural resources while minimizing the negative impact on the environment. Information on crops, rangeland, livestock, and other agricultural resources is critical for effective management of depleting and scarce resources. The implementation of the PA involves the integration of smart technologies in both farming and livestock, allowing the farmer to manage field variability to maximize the cost–benefit ratio and also to continuously and/or automatically monitor the main animal performance indicators.

This literature review allowed us to realize that PA has been proven to be a highly researched and constantly evolving area due to the needs of farmers to use resources in a more optimized way. Many techniques presented in this study were applied under strict controlled conditions for research and their implementation on the farms would also require some ability for auto-calibration of the device or tools. The development and operation of PA applications involve several steps and techniques that need to be investigated further to make the developed systems accurate and implementable in commercial environments and diverse farming systems. In addition, future research should also focus on the current need for farmers to acquire more knowledge regarding PA, as this may be one of the main factors dissuading them from implementing smart technologies in their fields.

## Data Availability

Not applicable.
